# Kinder im Blick? Kindeswohl in Zeiten von Corona

**DOI:** 10.1007/s12054-020-00315-1

**Published:** 2020-09-03

**Authors:** Birgit Jentsch, Brigitte Schnock

**Affiliations:** Abteilung Familie und Familienpolitik, Fachgruppe Frühe Hilfen, Deutsches Jugendinstitut e. V., München, Deutschland

**Keywords:** Corona, Kindeswohl in Pandemien, Telefonische und digitale Kommunikation, Face-to-face-Kontakte

## Abstract

Die Corona-Krise hat große Auswirkungen auf das Wohlergehen von Kindern sowie auf die Möglichkeiten von Fachkräften der Kinder- und Jugendhilfe, Familien im Bedarfsfall ausreichend zu unterstützen. Es häufen sich Anzeichen, dass insbesondere die Wahrnehmung von Kindeswohlgefährdung und die Gestaltung der Kommunikation und Beziehung zwischen Familie und Fachkraft erhebliche neue Herausforderungen stellen. Der Artikel beschreibt den Diskurs über diese Herausforderungen und Umsetzungsstrategien in den ersten Wochen von COVID-19.

Während Kinder in Zeiten von Pandemien erhöhten Risiken von Misshandlung und Vernachlässigung ausgesetzt sind, kann präventiver und intervenierender Kinderschutz vielfach nicht mehr durchgängig und im geeigneten Maße gewährleistet werden. Behörden und Fachkräfte der Kinder- und Jugendhilfe sind durch handlungsbeschränkende Infektionsschutzmaßnahmen vor erhebliche Herausforderungen gestellt. Wie können Kinder vor Gefahren für ihr Wohl geschützt werden, wenn unterstützende Akteur_innen in Schulen, Kitas, Artzpraxen und Vereinen in ihren Handlungsmöglichkeiten eingeschränkt sind? Wie kann es kurzfristig gelingen, Face-to-face-Kontakte weitgehend auf digitale und telefonische Kontakte umzustellen? Und wie können Face-to-face-Kontakte, wo sie notwendig bleiben, gestaltet werden? Kreative Lösungsansätze sind gefragt.

Für schutzbedürftige Kinder ist es in Zeiten von Pandemien besonders wichtig, dass die Kontinuität ihrer Versorgung sichergestellt ist (Sistovaris et al. 2020, S. 11). Erste Rückmeldungen der wichtigsten Akteure wie Familien, Sozialarbeiter_innen und Fachverbände deuten jedoch darauf hin, dass die relevanten Systemressourcen und -kapazitäten vor großen Herausforderungen stehen (Sistovaris et al. 2020). Diese werden dadurch weiter verschärft, dass Pandemien die Lebenslagen von Familien sowohl direkt als auch indirekt nachteilig beeinflussen können. Die direkten Risiken bestehen in Krankheit und Tod, den damit einhergehenden Veränderungen im Familiengefüge und daraus resultierenden psychosozialen und wirtschaftlichen Belastungen (The Alliance 2019a). Zu den indirekten Auswirkungen gehört die Zunahme von Existenzängsten und Konflikten in den Familien (Kelly und Hansel 2020; Sistovaris et al. 2020; The Alliance 2019a, 2019b). Familien in prekären Lebenssituationen gelten als besonders gefordert, gefährdet und auf professionelle Unterstützung der Kinder- und Jugendhilfe angewiesen (The Alliance 2019b).

## Zahlen und Indikatoren zum erhöhten Gefährdungsrisiko

In Großbritannien gab es schon früh aussagekräftige Zahlen zum Risiko familialer Gewalt in Zeiten von Corona: In den ersten Wochen nach der Ausgangssperre wurden mindestens 16 Frauen und Kinder zu Hause getötet, fünf sind es in ‚normalen‘ Zeiten (Smith 2020). In Deutschland lieferte die 6. Welle des COVID-19-Snapshot-Monitorings Anfang April 2020 frühe Ergebnisse zur Entwicklung häuslicher Gewalt: Selbst-Quarantäne, Gefühle von Niedergeschlagenheit und Sorge um den Arbeitsplatz erhöht die Wahrscheinlichkeit von (auch körperlichen) Konflikten in Ehe und Partnerschaft zum Teil ganz erheblich (Betsch et al. 2020; vgl. auch Steinert und Ebert 2020).

Ansonsten waren es in den ersten Wochen der Kontaktbeschränkungen vor allem Indikatoren, auf denen in Deutschland die Annahme eines erhöhten Risikos für Kindeswohlgefährdung gründete, wie die Zunahme der Beratungen am Elterntelefon „Nummer gegen Kummer“ und der Chatberatung für Kinder und Jugendliche (BMFSFJ 2020). Allerdings wurde zu diesem Zeitpunkt auch auf erste Rückmeldungen von Beratungsstellen verwiesen, denenzufolge die Zahl der Mitteilungen wegen Verdacht auf Kindeswohlgefährdung eher rückläufig sind (Der Tagesspiegel 2020; vgl. international auch Smith 2020; NGA 2020). Dies zeigte auch eine Umfrage der Süddeutschen Zeitung und des WDR bei deutschen Jugendämtern (Hell et al. 2020). Die medizinische Kinderschutzhotline für Angehörige von Gesundheitsberufen dagegen vermeldete für die ersten beiden Mai-Wochen einen drastischen Anstieg an Anrufen, die sich auf 50 zum Teil schwere Verdachtsfälle bezog und damit auf fast so viele Fälle wie im gesamten April. Betroffen seien besonders Kleinstkinder (Der Tagesspiegel 2020).

## Strukturelle Hintergründe eines erhöhten Risikos von Misshandlung oder Vernachlässigung

Bei der Frage nach den möglichen Ursachen eines höheren Risikos von Misshandlung oder Vernachlässigung bei Kindern und Jugendliche in Zeiten von COVID-19 rückt die große Bedeutung von Schulen und Kitas, Kindertagespflege, Freizeitangebote (z. B. in Sportvereinen) und Pädiatrie für den Kinderschutz in den Blick: Etwa 40 % der Gefährdungsmitteilungen kommen normalerweise von Schulen, Kitas, Kinderarztpraxen u. a. (Autorengruppe Kinder und Jugendhilfestatistik 2019, S. 139), die mit Corona allerdings geschlossen bzw. wegen Infektionsängsten weniger aufgesucht werden.

Vor diesem Hintergrund wiegt es aus Expertensicht umso schwerer, dass es in Deutschland nicht überall und auf Anhieb gelang, Notbetreuung in Kitas und Schulen sicherzustellen und bundeseinheitlich zu regeln (Die Kinderschutz-Zentren 2020), insbesondere auch für vulnerable Kinder (Deutsche Gesellschaft für Psychologie 2020). Umso problematischer ist es, wenn Maßnahmen, die unmittelbar für den Kinderschutz bedeutsam sind, mit Corona nur noch reduziert zum Tragen kommen. So berichteten zum Beispiel in einer Umfrage des Bayrischen Rundfunks bei 14 bayrischen Jugendämtern Anfang Mai die Behörden, dass Hausbesuche zum Teil wegfallen und die Fachkräfte nur noch in Notfällen, nämlich bei akuter Kindeswohlgefährdung, im Außendienst sind, und ansonsten Kontakt zu den Familien nur noch über Telefon, E‑Mail und Video-Chat pflegen (Hell et al. 2020).

## Mitteilungen von Fällen

Da für wichtige Akteur_innen im Kinderschutz (z. B. Lehrer_innen und Erzieher_innen) unter den Bedingungen von Distanzgeboten ein regelmäßiger Kontakt zu den Kindern kaum mehr möglich ist, wurde eine Vielzahl von Maßnahmen und Strategien entwickelt, mit denen die breite Öffentlichkeit proaktiv in den Schutz des Kindeswohl einbezogen, und belastete oder unter Druck geratene Familien erreicht werden sollen: Über Internetseiten, Flyer, Infomails und WhatsApp-Nachrichten an Adressat_innen werden Zugänge zu Hilfe- und Beratungsangeboten offensiv beworben; teilweise sind Informationen gezielt an Nachbarschaft, Verwandte und Bekannte von Familien sowie an Fachkräfte im Gesundheitswesen gerichtet, wie sie Familien bei der Suche nach Hilfe unterstützen und bei Verdacht auf Kindeswohlgefährdung vorgehen können. Auf internationaler Ebene empfiehlt The Alliance (2020), Akteure zum Schutz des Kindeswohls zu involvieren, die trotz Abstandsgeboten Kontakt zu Kindern haben können, wie z. B. Apotheker_innen und Einzelhändler_innen (The Alliance 2020 S. 3). Apotheken in Spanien, Frankreich und Großbritannien unterstützen Opfer häuslicher Gewalt, die im Lockdown nicht in der Lage sind, zu Hause in Gegenwart des Täters Hilfe zu suchen (Elks und Davies 2020; Grierson 2020). In Deutschland wurde vom Bundesministerium die Initiative „Stärker als Gewalt“ ins Leben gerufen, in deren Rahmen Supermärkte mithilfe von Plakaten im Geschäftsbereich und auf der Rückseite von Kassenzetteln über Hilfeangebote bei häuslicher Gewalt informieren.

## Die Bedeutung persönlicher Kontakte in Krisenzeiten

Der Tenor in einer Vielzahl von Stellungnahmen ist von Beginn der Kontaktbeschränkungen an: Persönliche Kontakte, gerade im Rahmen der aufsuchenden Arbeit im Kinderschutz, müssen auch unter den Krisenbedingungen von Corona weiterhin gepflegt und sogar ausgebaut werden. Als eine der größten Herausforderungen stellt sich hierbei dar, innovative – und gerade auch digitale – Wege zu finden, um junge Menschen und Familien zu unterstützen und zu begleiten (Bundesjugendkuratorium 2020).

## Telefonische und digitale Beratung und Begleitung der Familien

Erfahrungsberichte aus der Praxis zeigen allerdings auf, dass es im telefonischen Kontakt deutlich erschwert sein kann, Beziehung aufzubauen, weil nonverbale Hinweise, Blicke und Augenkontakt fehlen (Community Care 2020). Auch bei diagnostischen Fragestellungen oder akuter Krisenintervention stößt Online- und telefonische Beratung an Grenzen (Reindl und Engelhardt 2020). Ein Vorteil von Video- gegenüber Telefonkontakten ist die Möglichkeit, Sicherheit und Wohlbefinden der Familie besser einschätzen und interpersonelle Signale geben und verstehen zu können (Waters et al. 2020). Besondere Herausforderungen bestehen allerdings, wenn es darum geht, mit (Klein‑)Kindern digital in Kontakt zu treten, oder mit Kindern und Eltern mit eingeschränkter Aufmerksamkeitsspanne oder mit verminderter Intelligenz (Reindl und Engelhardt 2020).

Einer qualitativen Ad-hoc-Befragung des Nationalen Zentrums Frühe Hilfen (NZFH) zufolge betreuen in Zeiten der Pandemie mehr als die Hälfte der Familienhebammen und Familienkrankenschwestern Schwangere und Eltern telefonisch, knapp ein Sechstel der Fachkräfte bleibt per E‑Mail, Videotelefonie oder Messengerdienst mit den Familien in Kontakt. Eine intensive Begleitung der Familien, wie sie in der aktuellen Krisensituation notwendig wäre, ist aus der Sicht der Fachkräfte allerdings nicht möglich (NZFH 2020).

## Herausforderungen der Digitalisierung von Arbeitsprozessen in der Jugendhilfe

Dem Bundesforum Vormundschaft und Pflegschaft (2020) zufolge scheinen sich Behörden – nach bisher eher kritischer Haltung kommerziellen Kommunikationsdiensten gegenüber – zunehmend für diese zu öffnen. Aus dem Bereich der Psychotherapie kommen Hinweise, dass auch auf der Ebene der Fachkräfte – selbst bei geringer Erfahrung in Sachen Videokonferenzen – der Kommunikationsstil schnell an diese Technik angepasst wird (Martin et al. 2020, S. 38 f.).

Wichtige Überlegungen zur Implementierung des digitalen Fallmanagements kommen vom Department of Health & Human Services in den USA. Drei Schritte seien hierzu erforderlich:

- Identifizierung geeigneter Technologie,
- Bereitstellung von Schulungen für Fachkräfte und Klientinnen und Klienten sowie
- Berücksichtigung von Datenschutz und Informationsaustausch zwischen Systemen (Waters et al. 2020).

Digitale Kommunikation kann über Plattformen erfolgen, über Videokonferenzen oder andere webbasierte Technologien, teilweise mit dem Potenzial eines virtuellen Fallverwaltungssystems auch für kinderschutzrelevante Organisationen. Bei der Auswahl der Technologie zu beachtende Faktoren sind u. a. die IT-Infrastruktur der Behörde bzw. des Trägers, die Komplexität der zu übermittelnden Informationen (Waters et al. 2020) sowie der Datenschutz.

Fachkräfte in der Jugendhilfe scheinen für Videokommunikation oft nicht ausreichend qualifiziert zu sein, sodass ein erheblicher Bedarf an Knowhow für digitalisiertes Arbeiten besteht. Dieser wurde zum Beispiel zeitnah in Webinaren des Deutschen Instituts für Jugendhilfe und Familienrecht e. V. für Jugendamtsleitungen und Beschäftigte im ASD zu decken versucht. Zudem gibt es inzwischen etliche Arbeitshilfen für Fachkräfte in der (Krisen‑)Beratung, auch speziell im Kinderschutz, in denen es um den Umgang mit Kommunikationstechnologie geht, aber auch um spezifische Kommunikationstrategien am Telefon oder im Videochat (Wenzel et al. 2020).

Bei allen Überlegungen, zur Reduzierung der Ansteckungsgefahr mit den Ratsuchenden online in Kontakt zu treten, darf allerdings nicht vergessen werden: Für viele Klientinnen und Klienten ist eine online-Beratung sehr hochschwellig – wegen sprachlicher und/oder kognitiver Barrieren oder weil der Zugang zum Netz fehlt. Längst nicht alle Familien verfügen über die notwendigen technischen Voraussetzungen für digitale Kommunikation, wie im Kontext des Homelearning sehr deutlich wurde. Dies ist auch in der Kinder- und Jugendhilfe mitzudenken.

## Datenschutzrechtliche Herausforderungen

Mit dem Einsatz digitaler Kommunikationsmedien stellt sich die Frage, wie der Datenschutz gewährleistet werden kann. Öffentliche und freie Träger verarbeiten vielfach personenbezogene Daten, die durch Datenschutzbestimmungen geschützt sind.

In Deutschland aber wird es teilweise für zulässig erachtet, in Anbetracht der Krisensituation durch Corona datenschutzrechtliche Fragen in den Hintergrund zu drängen und die – unumgängliche – Klärung datenschutzrechtlicher Fragen auf die Zeit der Normalisierung der Lage zu verschieben (AGJ 2020, S. 4). So wurden etwa in Bayern vorübergehend Lockerungen des Datenschutzes seitens des Landesdatenschutzbeauftragten – vorerst gültig bis zum 14.06.2020 (Stand 27.05.2020) – bekanntgegeben, die die Verwendung von Privatgeräten sowie die Nutzung von Messengern und Clouddiensten von Beschäftigten in öffentlichen Stellen untereinander sowie mit Personen außerhalb öffentlicher Einrichtungen zulassen (Der Bayerische Landesbeauftragte für den Datenschutz 2020).

Allerdings gibt es seitens verantwortlicher Stellen inzwischen eine Vielzahl von Informationen, wie Datenschutz bei verstärkter digitaler Kommunikation gewährleistet werden kann (Reindl und Engelhardt 2020). Einschlägige Empfehlungen finden sich auch auf der Website des Bundesbeauftragten für den Datenschutz und die Informationsfreiheit, und zwar zur Nutzung von Messenger- und Videokonferenzdiensten in Zeiten der Pandemie sowie in Bezug auf die kompetente Beurteilung von digitalen Angeboten (www.bfdi.bund.de/DE/Datenschutz/Datenschutz-Corona/Kommunikation/Kommunikations-node.html). Datenschutzrechtliche Fragen werden außerdem auch in den FAQs des DIJUF behandelt (https://www.dijuf.de/coronavirus-faq.html#finFAQ4).

## Face-to-face-Kontakte

Face-to-face-Kontakte mit Familien stellen einen integralen Bestandteil der Sozialarbeit im Kinderschutz dar, z. B. bei Hausbesuchen, bei Beratungs- und Hilfeplangesprächen. Diese persönlichen Begegnungen werden zumindest in ausgewählten Fällen auch während einer Pandemie fortgeführt. Manche Fachkräfte haben sich während der Corona-Krise, z. B. bei Bestehen eines Schutzplans, mit Familien getroffen. Gebotene Abstände und Hygieneschutzregeln wurden dabei gewahrt und neue bzw. variierte Settings genutzt, wie z. B. Treffen im Freien (Berrischen 2020). Verunsicherungen der Eltern, inwieweit sie in Zeiten der Kontaktbeschränkung eine persönliche Begegnung mit Fachkräften zulassen können, wurden in der Praxis z. B. mit einem Elternbrief begegnet, der darauf abzielt, diese Ängste aufzugreifen und zu einer gemeinsamen Lösung zu kommen (Forum Transfer o.J.). Allerdings kann die elterliche Formulierung von Angst vor einer Ansteckung insbesondere bei unfreiwilligen Klientinnen und Klienten auch ein Vorwand sein um einen persönlichen Kontakt zu verhindern (DIJuF 2020).

Was den Schutz von Fachkräften betrifft, ziehen manche Jugendämter bei Verdacht auf eine Corona-Infektion bei Klient_innen einen Verzicht auf Hausbesuche vor, zugunsten einer Unterstützung per Telefon, E‑Mail oder Videochat (Forum Transfer o.J.). Ein privater Träger in England empfiehlt seinen Fachkräften, vor der Entscheidung für ein Face-to-face-Treffen einen Fragebogen durchzugehen. Dieser geht auch auf den Schutz von Risikogruppen ein (ob Klient_innen oder Fachkraft Vorerkrankungen haben oder über 70 Jahre alt sind), sowie auf Risiken bei der Anfahrt zu den Familien, z. B. mit öffentlichen Verkehrsmitteln (WillisPalmer 2020).

Darüber hinaus verweist die International Federation of Social Workers (IFSW) auf ihre ethischen Grundsätze, wonach Sozialarbeiter_innen nicht zu einer gesundheitsgefährdenden Arbeit gezwungen werden können (Wehrmann 2020, S. 4). Gleichzeitig wird auf Vorsichtsmaßnahmen der WHO hingewiesen, wie man sich vor dem Virus schützen kann. Die IFSW fordert dabei, dass aufsuchende Sozialarbeit ausreichend mit Schutzkleidung versorgt wird, inklusive geeigneter Masken und Handschuhe (Wehrmann 2020). Ähnliche Forderungen stellen deutsche Verbände und Gewerkschaften: In der Phase der akuten Krisenbewältigung seien in Deutschland zunächst die Bedarfe in Gesundheitswesen und Pflege priorisiert worden, andere systemrelevante Akteure seien im Hintergrund geblieben. Diese benötigten nun auch dringend finanzielle und materielle Unterstützung (Der Paritätische Gesamtverband 2020).

Die AGJ (2020, S. 3) berichtet jedoch von offenen Fragen bezüglich nicht vorhandener Schutzkleidung bei notwendigen Hausbesuchen. Dies fügt sich in den internationalen Diskurs ein, in dem wiederholt erwähnt wird, wie Sozialarbeiter_innen wegen Mangel an Schutzkleidung selbst kreativ werden müssen. So hat eine Ausschussvorsitzende der International Association of Schools of Social Work (IASSW) eine Liste von improvisierten Schutzkleidungsartikel erstellt, die wegen mangelnder Alternativen genutzt werden müssen (z. B. Ski- anstelle von Schutzbrillen; Müllsäcke anstelle von Overalls) (Dominelli 2020, S. 7).

In der internationalen Literatur wird auch empfohlen, zusammen mit den Communities, in die Familien eingebettet sind, Strategien zu entwickeln, um vulnerable Familien in Zeiten von Pandemien zu unterstützen. Zu den vertrauenswürdigen Erwachsenen, die Kontakt mit Eltern und Kinder aufnehmen können, zählen pensionierte Sozialarbeiter_innen sowie Menschen, die bereits über ein polizeiliches Führungszeugnis verfügen – von beurlaubten Erzieher_innen bis zu Sporttrainer_innen (The Alliance 2019b; Welch und Haskins 2020; Simpson 2020).

## Ausblick

Obwohl die schrittweise Öffnung des Alltagslebens auch vermehrt persönliche Kontakte ermöglicht, bleibt die Corona-Krise dynamisch. Potenzielle weitere Infektionswellen erfordern Strategien, die auf den nationalen und internationalen Erfahrungen mit verschiedenen Lösungsansätzen aufbauen sollten. Ein wichtiges Thema wird hier die systematische Nutzung sozialer Medien in der Beratung der Familien durch die Fachkräfte sein. Gerade in diesen Kontexten können solche Medien ein enormes Potential entfalten, da Fachkräfte es gewohnt sind, über das Medium Sprache oder Schrift komplexe Inhalte zu beraten. Den momentanen Schub, sich an solche Medien verstärkt heranzutrauen und sie inhaltlich zu nutzen, gilt es in nachhaltige Strukturen zu überführen.

Die Pandemie hat aber auch mit den alternativen Beratungsformen, die zurzeit ergänzend zu weiterhin unerlässlichen persönlichen Kontakten erprobt und umgesetzt werden, einer lange überfälligen Entwicklung Vorschub geleistet. Diese Entwicklung gilt es auch nach der Krise konsequent fortzuführen und Fachkräfte in der Kinder- und Jugendhilfe mit aller hierfür notwendigen Technik auszustatten und entsprechend zu schulen. Auch in „Normalzeiten“ können solche Beratungssettings das Beratungsspektrum erweitern und niedrigschwellige Zugänge für die verschiedens Zielgruppen sicherstellen, selbst wenn ihr Nutzen beim Kinderschutz Grenzen hat.

## References

[1] AGJ (2020). Wenn Kümmerer*innen selbst Hilfe brauchen… Die Auswirkungen der Corona-Krise auf die Kinder- und Jugendhilfe, Zwischenruf der Arbeitsgemeinschaft für Kinder- und Jugendhilfe. AGJ, 27.3.2020. https://www.agj.de/fileadmin/files/positionen/2020/AGJ_Zwischenruf_Corona.pdfZugegriffen: 25. Aug. 2020.

[2] Autorengruppe Kinder- und Jugendhilfestatistik (2019). Kinder- und Jugendhilfereport 2018. Eine kennzahlenbasierte Analyse.

[6] Berrischen, B. (2020). Familienberatung im Rhein-Kreis: Beratungsstellen trotz Corona erreichbar. NGZ online, 02.04.2020. https://rp-online.de/nrw/staedte/rhein-kreis/familienberatungsstellen-im-rhein-kreis-trotz-corona-krise-erreichbar_aid-49784475. Zugegriffen: 25. Aug. 2020.

[7] Betsch C (2020). COVID-19 Snapshot Monitoring (COSMO) – Welle 6 Ergebnisse aus dem wiederholten querschnittlichen Monitoring von Wissen, Risikowahrnehmung, Schutzverhalten und Vertrauen während des aktuellen COVID-19 Ausbruchsgeschehens. Stand: 11.04.2020 (Version 06-02).

[10] BMFSFJ (2020). Schutz von Kindern und Jugendlichen vor häuslicher Gewalt. Ministerin Giffey stimmt sich mit Ländern über Maßnahmen in der Corona-Krise ab. Pressemitteilung vom 31.3.2020. https://www.bmfsfj.de/bmfsfj/aktuelles/presse/pressemitteilungen/schutz-von-kindern-und-jugendlichen-vor-haeuslicher-gewalt/154262. Zugegriffen: 25. Aug. 2020.

[8] Bundesforum Vormundschaft und Pflegschaft (2020). Vormundschaft in Zeiten der Corona-Krise. Hinweise aus der Praxis für die Praxis. https://vormundschaft.net/vormundschaft-in-zeiten-der-corona-krise/. Zugegriffen: 25. Aug. 2020.

[9] Bundesjugendkuratorium (2020). Zwischenruf des Bundesjugendkuratoriums BJK. Unterstützung von jungen Menschen in Zeiten von Corona gestalten! Kinder- und Jugendpolitik ist gefordert. 24.3.2020. https://bundesjugendkuratorium.de/stellungnahmen Zugegriffen: 25. Aug. 2020.

[11] Der Bayerische Landesbeauftragte für den Datenschutz (2020). Sonderinformationen zum mobilen Arbeiten mit Privatgeräten zur Bewältigung der Corona-Pandemie. https://www.datenschutz-bayern.de/corona/sonderinfo.html. Zugegriffen: 25. Aug. 2020.

[36] CommunityCare (2020). Social work diary: ‵I miss the non-verbal cues, the looks and the eye-contact.‵ 30.04.2020. https://www.communitycare.co.uk/2020/04/30/social-work-diary-i-miss-the-non-verbal-cues-the-looks-and-eye-contact/. Zugegriffen: 26.08.2020.

[24] Der Paritätische Gesamtverband (2020). Bonus für soziale Arbeit während Corona-Krise: Paritätischer fordert Steuerabzug für alle Beschäftigten in sozialen Diensten in Höhe von 500 Euro. Pressemeldung vom 09.04.2020. https://www.der-paritaetische.de/presse/bonus-fuer-soziale-arbeit-waehrend-corona-krise-paritaetischer-fordert-steuerabzug-fuer-alle-beschaeftig/. Zugegriffen: 25. Aug. 2020.

[12] Der Tagesspiegel (2020). „Knochenbrüche oder Schütteltraumata“. Mediziner berichten von massiver Gewalt gegen Kinder. 15.5.2020. https://www.tagesspiegel.de/politik/knochenbrueche-oder-schuetteltraumata-mediziner-berichten-von-massiver-gewalt-gegen-kinder/25833740.html. Zugegriffen: 25. Aug. 2020.

[13] Deutsche Gesellschaft für Psychologie (2020). Stellungnahme zur Notwendigkeit der Notbetreuung von psychisch kranken Kindern und Kindern von psychisch kranken Eltern in Kindertagesstätten und Schulen während der Coronakrise. 31.3.2020. https://www.dksb.de/fileadmin/user_upload/DGPs-Stellungnahme_CoronaNotbetreuung_20203103-2.pdf. Zugegriffen: 25. Aug. 2020.

[15] Die Kinderschutz-Zentren (2020). Was Kinderschutz in der gegenwärtigen Krise bedeutet und braucht. Ein Zwischenruf der Kinderschutzzentren. 1.4.2020. https://www.kinderschutz-zentren.org/Mediengalerie/1585749029_-_Zwischenruf_der_Kinderschutz-Zentren.pdf. Zugegriffen: 25. Aug. 2020.

[14] DIJuF (2020). Wie ist damit umzugehen, wenn eine Familie einen im Rahmen einer (möglichen) Kindeswohlgefährdung notwendigen Hausbesuch mit Verweis auf die Infektionsgefahr verweigert? (Stand: 16.4.2020). https://www.dijuf.de/coronavirus-faq.html#kindFAQ3. Zugegriffen: 25. Aug. 2020.

[16] Dominelli L (2020). Surviving Covid-19: social work issues in a global pandemic (child protection and welfare, social care).

[17] Elks, S., & Davies, S. (2020). Coronavirus codewords: help or hindrance in domestic abuse? Thomas Reuters Foundation News. https://news.trust.org/item/20200415175930-b0dk. Zugegriffen: 25. Aug. 2020.

[37] Forum Transfer. Innovative Kinder- und Jugendhilfe in Zeiten von Corona (o. J.). Umgang mit Unsicherheiten. https://www.forum-transfer.de/handlungsfelder/hilfen-zur-erziehung/ ambulante-hilfen/umgang-mit-unsicherheiten.html. Zugegriffen: 26.08.2020.

[19] Grierson, J. (2020). Boots to provide help for domestic abuse victims. The Guardian, 01.05.2020. https://www.theguardian.com/business/2020/may/01/boots-to-provide-help-for-domestic-abuse-victims-coronavirus-lockdown. Zugegriffen: 25. Aug. 2020.

[20] Hell, A., Kampf, L., Kaulet, M., et al. (2020). Häusliche Gewalt in der Corona-Krise. Wenn das Kind verborgen bleibt. Süddeutsche Zeitung, 6. Mai 2020. https://www.sueddeutsche.de/politik/coronavirus-haeusliche-gewalt-jugendaemter-1.4899381. Zugegriffen: 25. Aug. 2020.

[34] Kelly, J., & Hansel, K. (2020) Coronavirus: What Child Welfare Systems Need to Think About. The Imprint. Youth And Family News, 11.03.2020. https://imprintnews.org/child-welfare-2/coronavirus-what-child-welfare-systems-need-to-think-about/41220. Zugegriffen: 26.08.2020.

[22] Martin J, McBride T, Masterman T (2020). Covid-19 and early intervention: Evidence, challenges and risks relating to virtual and digital delivery.

[35] National Governors Association (NGA) (2020). Addressing child abuse reporting and supporting child well-being during Covid-19. 15.06.2020. https://www.nga.org/memos/child-abuse-reporting-covid-19/. Zugegriffen: 26.08.2020.

[23] NZFH (2020). Befragung von Gesundheitsfachkräften zu den Veränderungen durch Corona. https://www.fruehehilfen.de/forschung-im-nzfh/forschung-zu-corona/befragung-von-gesundheitsfachkraeften-zu-den-veraenderungen-durch-corona/. Zugegriffen: 25. Aug. 2020.

[21] Reindl R, Engelhardt E (2020). Handlungsempfehlungen zur kurzfristigen Umsetzung von Onlineberatung vor dem Hintergrund der Corona-Krise. Institut für E-Beratung der Technischen Hochschule Nürnberg.

[25] Simpson, F. (2020). BASW makes fresh plea for PPE as social worker dies from Covid-19. Children & Young People Now. 01.04.2020. https://www.cypnow.co.uk/news/article/basw-makes-fresh-plea-for-ppe-as-social-worker-dies-from-covid-19. Zugegriffen: 25. Aug. 2020.

[26] Sistovaris M, Fallon B, Miller S (2020). Child welfare and pandemics.

[27] Smith, K. I. (2020). Coronavirus doesn’t cause men’s violence against women. https://kareningalasmith.com/2020/04/15/coronavirus-doesnt-cause-mens-violence-against-women/. Zugegriffen: 25. Aug. 2020.

[28] Steinert, J., & Ebert, C. (2020). Gewalt an Frauen und Kindern in Deutschland während COVID-19-bedingten Ausgangsbeschränkungen: Zusammenfassung der Ergebnisse. Hochschule für Politik München (HfP) und der TUM School of Governance. https://www.gov.tum.de/globalhealth/forschung/covid-19-and-domestic-violence/. Zugegriffen: 25. Aug. 2020.

[4] The Alliance for Child Protection in Humanitarian Action (2019a). Minimum Standards for Child Protection in Humanitarian Action. The Alliance for Child Protection in Humanitarian Action, Minimum Standards for Child Protection in Humanitarian Action. 2019 Edition. https://handbook.spherestandards.org/en/cpms/#ch001. Zugegriffen: 25. Aug. 2020.

[5] The Alliance for Child Protection in Humanitarian Action (2019b). Technical Note: Protection of Children during the Coronavirus Pandemic. Version 1, March 2019. https://alliancecpha.org/system/tdf/library/attachments/the_alliance_covid_19_brief_version_1.pdf?file=1&type=node&id=37184. Zugegriffen: 25. Aug. 2020.

[3] The Alliance for Child Protection in Humanitarian Action (The Alliance) (2020). End Violence Against Children, UNICEF, WHO, COVID-19: Protecting Children from Violence, Abuse and Neglect in the Home. Version 1, May 2020. https://www.unicef.org/media/68711/file/COVID-19-Protecting-children-from-violence-abuse-and-neglect-in-home-2020.pdf. Zugegriffen: 25. Aug. 2020.

[30] Waters, A., Winston, P., & Ghertner, R. (2020). Virtual Case Management Considerations and Resources for Human Services, US Department of Health & Human Services, 01.04.2020. https://aspe.hhs.gov/pdf-report/virtual-case-management. Zugegriffen: 25. Aug. 2020.

[31] Wehrmann, C. (2020). Ethical decision-making in the face of COVID-19. International Federation of Social Workers (IFSW). https://www.ifsw.org/wp-content/uploads/2020/04/Option-A-Ethical-Decision-making-in-the-face-of-COVID-19.pdf. Zugegriffen: 25. Aug. 2020.

[32] Welch, M., & Haskins, R. (2020). Report. What COVID-19 means for America’s child welfare system, Brookings, 30.04.2020. https://www.brookings.edu/research/what-covid-19-means-for-americas-child-welfare-system/. Zugegriffen: 25. Aug. 2020.

[33] Wenzel, J., Jaschke, S., & Engelhardt, E. (2020). Handreichung. Krisenberatung am Telefon und per Video in Zeiten von Corona. Aktualisiert: 08.04.2020, Version: 1.2. https://www.fruehehilfen.de/fileadmin/user_upload/fruehehilfen.de/pdf/Krisenberatung-am-Telefon-und-per-Video-in-Zeiten-von-Corona-2020-03-27-VS-1-1.pdf. Zugegriffen: 25. Aug. 2020.

[38] WillisPalmer (2020) Updated Guidance - Covid 19. https://www.willispalmer.com/wp-content/uploads/2020/06/Updated-Guidance-Covid-19-11.6.20.pdf. Zugegriffen: 26.08.2020.

